# Negative emotionality shapes the modulatory effects of ketamine and lamotrigine in subregions of the anterior cingulate cortex

**DOI:** 10.1038/s41398-024-02977-x

**Published:** 2024-06-18

**Authors:** Matti Gärtner, Anne Weigand, Marvin Sören Meiering, David Weigner, Luisa Carstens, Christian Keicher, Rita Hertrampf, Christian Beckmann, Maarten Mennes, Andreas Wunder, Simone Grimm

**Affiliations:** 1 Medical School Berlin, Berlin, Germany; 2 Charité Research Organisation GmbH, Berlin, Germany; 3 SBGneuro Ltd., Oxford, UK; 4 Translational Medicine and Clinical Pharmacology, Boehringer Ingelheim Pharma GmbH & Co. KG, Biberach an der Riss, Germany; 5 Department of Psychiatry and Psychotherapy, Charité, Universitätsmedizin Berlin, Corporate Member of Freie Universität Berlin and Humboldt-Universität Zu Berlin, Berlin, Germany; 6 Department of Psychiatry, Psychotherapy and Psychosomatics, University Hospital of Psychiatry, University of Zurich, Zurich, Switzerland

**Keywords:** Predictive markers, Molecular neuroscience

## Abstract

Neuroimaging studies have identified the anterior cingulate cortex (ACC) as one of the major targets of ketamine in the human brain, which may be related to ketamine’s antidepressant (AD) mechanisms of action. However, due to different methodological approaches, different investigated populations, and varying measurement timepoints, results are not consistent, and the functional significance of the observed brain changes remains a matter of open debate. Inhibition of glutamate release during acute ketamine administration by lamotrigine provides the opportunity to gain additional insight into the functional significance of ketamine-induced brain changes. Furthermore, the assessment of trait negative emotionality holds promise to link findings in healthy participants to potential AD mechanisms of ketamine. In this double-blind, placebo-controlled, randomized, single dose, parallel-group study, we collected resting-state fMRI data before, during, and 24 h after ketamine administration in a sample of 75 healthy male and female participants who were randomly allocated to one of three treatment conditions (ketamine, ketamine with lamotrigine pre- treatment, placebo). Spontaneous brain activity was extracted from two ventral and one dorsal subregions of the ACC. Our results showed activity decreases during the administration of ketamine in all three ACC subregions. However, only in the ventral subregions of the ACC this effect was attenuated by lamotrigine. 24 h after administration, ACC activity returned to baseline levels, but group differences were observed between the lamotrigine and the ketamine group. Trait negative emotionality was closely linked to activity changes in the subgenual ACC after ketamine administration. These results contribute to an understanding of the functional significance of ketamine effects in different subregions of the ACC by combining an approach to modulate glutamate release with the assessment of multiple timepoints and associations with trait negative emotionality in healthy participants.

## Introduction

The effects of ketamine on the human brain have been investigated in several neuroimaging studies over the past decades [1]. The renewed interest in ketamine is closely linked to the discovery of its rapid antidepressant (AD) properties [2] that have been considered a crucial paradigm shift in recent depression research [3]. Neuroimaging studies in healthy volunteers and depressed patients have identified important targets for ketamine in the human brain, but to date, the results do not converge and the functional significance of ketamine-induced brain changes remains largely unknown.

A recent review suggests that activity changes in the anterior cingulate cortex (ACC) after ketamine administration may be specifically linked to the AD mechanism of ketamine [4]. However, while numerous studies have repeatedly implicated different ACC subregions in ketamine-induced brain changes, the direction and functional significance of these effects remain unclear. Important reasons for the inconsistent results are differing imaging modalities and populations, as well as varying assessment timepoints after ketamine administration. Furthermore, independent of the study of ketamine-induced changes, ACC subregions have been associated with distinct functions. Converging evidence suggests that the ventral subregions (subgenual and pregenual; sgACC, pgACC) are predominantly involved in emotional processing, while the dorsal ACC (dACC) has mainly been implicated in cognitive control and the integration of cognition and emotion [5, 6]. Regarding these distinct functional roles, it could be assumed that the functional significance of ketamine effects in the ventral ACC subregions might be distinct from the effects in the dorsal ACC. However, to the best of our knowledge this has not been systematically investigated yet.

Several studies in healthy volunteers reported a rapid and focal decrease of activity in the sgACC during acute ketamine administration [7–10], which might suggest a straightforward AD mechanism of ketamine, as overactivity and normalization of sgACC activity have been associated with acute depression [11] and remission, respectively [12]. However, it remains an open question whether acute activity reductions in the sgACC are directly linked to the AD mechanism of ketamine. While Ballard et al. (2015) reported that decreased sgACC activity approximately 4 h after ketamine administration was linked to reductions in suicidal ideation in depressed patients, Downey et al. (2016) rather found an association between symptom reduction and increased sgACC activity during ketamine administration [13]. The pregenual (pgACC) and dorsal ACC (dACC) have also been repeatedly associated with ketamine effects [4]. However, pronounced activity changes directly after administration have been less consistently reported for these regions, even though the pgACC supposedly plays a key role in mediating antidepressant effects [14], and has been discussed as a multimodal neuroimaging biomarker of early treatment response to ketamine [15]. We previously reported increased pgACC activity in response to negative emotional stimuli 24 h after ketamine administration in healthy participants [16] as well as an association of pretreatment pgACC volume and activity with rapid AD effects of ketamine in patients [15, 17]. Depending on the investigated modality, rather inconsistent findings have also been reported for the dACC. Some studies reported increased dACC activity, and increased glutamate concentration after ketamine [9, 18, 19], yet another study found decreased cerebral blood flow in the dACC [20].

When interpreting the results of previous imaging studies, it is important to consider the timing of the measurement. Studies were usually either performed during acute ketamine administration or in a delayed time window—often 24 h after administration—depending on the research question. Ketamine induces an acute dissociative state that disappears shortly after the administration is discontinued [2]. Some studies have linked acute changes in the sgACC to the dissociative state [7, 8]. In the delayed time window (24 h), the subjective experience of dissociation has disappeared [21], and due to the short elimination half-life plasma concentration of ketamine is below an active threshold [22]. The main reason to study delayed effects is that the strongest AD effects of ketamine have been observed in this time window [23]. Interestingly, imaging studies in depressed patients have linked both, acute [13], and delayed [15, 24] brain changes after ketamine to the AD effects, and a theoretical model for multistage drug effects in which the acute effects are an important precursor to the delayed effects has been proposed [25]. Therefore it is important to investigate the interplay between acute and delayed effects of ketamine, but only very few imaging studies have included an acute and a delayed timepoint [26, 27].

Utilizing techniques to modulate glutamate transmission can offer additional insights into the mechanisms of action of ketamine. Accordingly, previous studies have demonstrated that the acute effects of ketamine on brain activity [7, 8, 28] and connectivity [29] were attenuated by lamotrigine, an anticonvulsant drug that inhibits glutamate release [30]. Attenuated dissociative symptoms [7, 31] underscored that the effects of acute ketamine challenge are sensitive to modulation by pretreatment with lamotrigine. However, Mathew et al. [32] and Abdallah et al. [33] found no effect of lamotrigine on the dissociative effects of ketamine.

As mentioned above, the renewed interest in ketamine’s mechanisms of action in the brain is closely linked to the discovery of its rapid AD properties. Patient studies have the obvious advantage that ketamine-induced brain changes can be directly linked to symptom improvement. However, the effects of disease progression and long-term medication can be major confounds [34]. Thus, studies in healthy subjects are useful to complement findings in patients, but it is important to consider that ketamine might have different effects on brain activity in healthy individuals and patients [4]. An approach that holds promise to link findings in healthy individuals to findings in patients is to adopt a dimensional approach by assessing trait negative emotionality in a healthy sample [34]. Previous research has provided compelling evidence that negative emotionality is a prevalent dimension of internalizing psychiatric disorders, and linked to the development of major depressive disorder [35]. Furthermore, it was recently hypothesized that ketamine might specifically act on neural systems associated with negative emotionality [36].

As previous studies have highlighted the importance of ACC in the neural mechanisms of ketamine, the overall aim of this study was to conduct a comprehensive investigation of this brain region during and after ketamine infusion. Specifically, our aims were to investigate the acute and delayed effects of ketamine in different subregions of the ACC. To investigate the effects of altered glutamate transmission, we also studied participants receiving lamotrigine before ketamine administration. To link the findings of the investigated healthy sample to the effects observed in patients, we examined the personality trait of negative emotionality in relation to ketamine effects.

## Methods

### Participants

Seventy-five healthy, right-handed male and female participants aged 18–45 years completed the fMRI procedures of this study. Before the participants were included in the study, the following exclusion criteria were queried and applied: A history of or current psychiatric conditions, as determined by the SCID-5-CV at screening, a positive drug screen, alcohol or substance dependence within the last 12 months, prescribed psychotropic medication within 28 days prior to screening and non-prescription medication within 48 h prior to treatment visit, a history of relevant neurological diseases, migraine headaches, relevant medical condition, MRI exclusion criteria, and pregnancy. All participants gave written consent to participate in the study, which was approved by the local ethics committee of the MSB Medical School Berlin and registered at ClinicalTrials.gov (NCT04156035). The study was conducted in compliance with Good Clinical Practices (ICH-GCP) and the Declaration of Helsinki, and in accordance with applicable legal and regulatory requirements. The progress of participant exclusion and inclusion is shown in the flow diagram in Supplementary Fig. 1.

### Experimental design and procedure

In this double-blind, placebo-controlled, randomized, single dose, parallel-group study with three treatment conditions, subjects meeting all in-/exclusion criteria were randomly assigned at baseline to one of three treatment groups in a 1:1:1 ratio after providing written informed consent. Subjects in the first group were pretreated with a placebo and were administered a placebo infusion (placebo-placebo group, PP). Subjects in the second group were pretreated with placebo and were administered a ketamine infusion (placebo-ketamine group, PK), and subjects in the third group were pretreated with lamotrigine and were administered ketamine (lamotrigine-ketamine group, LK). All subjects underwent two scanning sessions on two consecutive days. Prior to the first scanning session, subjects were pretreated with an oral dose of 300 mg lamotrigine (LK) or matching placebo (PP, PK) 2 h before they entered the scanner. The lamotrigine dosage was adopted from previous studies [7, 8] and was received in a fasting state. During the first scanning session (acute), subjects were intravenously administered racemic ketamine or placebo (ketamine dosage: 0.12 ± 0.003 mg/kg during the first minute, followed by a continuous infusion of ~0.31 mg/kg/h). The infusion paradigm was based on previous publications and intended to induce a rather low level of subjective effects, while eliciting a reliable response in the fMRI [8, 37]. Before the infusion started, all subjects underwent a short resting state scan, that was repeated after the start of the infusion. Afterward, the scanning procedures continued with two task-based fMRI sequences, and an ASL sequences that are reported elsewhere. Total scanning time was ~1 h. To investigate the possible delayed effects of a single dose of ketamine, subjects underwent the same scanning procedure without the drug treatment and without the baseline resting state scan 24 h later. A priori power analysis was conducted in G*Power [38] to determine a sufficient sample size using an alpha of 0.05 and a power of 0.8. Expected effect sizes were based on previous reports [39]. More details about the design and procedures are provided by Gärtner et al. [28].

### Materials

#### Psychometric assessments

Psychotomimetic and dissociative effects were assessed after each scanning session (acute and delayed timepoints) using the Dissociation-Tension-Scale (DSS) [40] and 5D Altered States of Consciousness Scale (5D-ASC) [41]. The DSS is a brief self-report measure of dissociative symptomology and consists of 22 items, which assess dissociative phenomena on a psychological, somatoform, and global scale. Psychological dissociative symptoms include depersonalization, derealization, or hallucinatory experiences. Somatoform dissociative symptoms include immobility and optical or acoustical perceptual changes. The 5D-ASC assesses altered states of consciousness with 94 items on five main dimensions: oceanic boundlessness (OBN), dread of ego dissolution (DED), visionary restructuralization (VRS), auditory alterations (AUA), and vigilance reduction (VIR). Participants use a visual analog scale to report the extent to which the experiences during the infusion differ from their normal waking state.

The German version of the Big Five Inventory 2 (BFI-2) [42] was used to assess trait negative emotionality. The BFI-2 consists of 60 items, rated on a five-point Likert scale, that determine five personality domains, i.e., extraversion, negative emotionality (alternatively labeled neuroticism), openness to experience, conscientiousness, and agreeableness [43]. Here we report the negative emotionality factor, which is expressing a trait experiencing elevated negative emotions like fear, anger, or sadness.

### MRI acquisition and analysis

Brain images were acquired using a 3 Tesla MRI scanner (PRISMA, Siemens Medical Systems, Erlangen, Germany) with a 20-channel head coil and a T2*- weighted gradient echo-planar imaging sequence of 6 min length (TR = 2 s, TE = 30 ms, flip angle = 80°, voxel size = 3 × 3 × 3 mm, matrix 64 × 64, 36 slices, FOV = 192 × 192 × 143 mm, GRAPPA acceleration factor 2). An anatomical brain image was acquired with a 3D T1-weighted scan (magnetization prepared rapid acquisition gradient echo sequence, TE = 3.03 ms, TR = 2.3 s, 192 slices, and FOV = 256 × 256 × 192 mm).

Resting-state fMRI data at the three-timepoints (baseline, acute, delayed) were analyzed in MATLAB (Version R2015a) using SPM12 and the CONN toolbox (Version 17c) [44]. Preprocessing of the fMRI data was performed using the default preprocessing pipeline “Direct normalization to MNI-space” available in CONN. The pipeline includes motion correction (realignment and unwarping), slice-timing correction, automatic detection of artifactual scans (ART-based scrubbing), normalization to MNI space, and spatial smoothing (8 mm). During the denoising step in CONN, single-subject linear regression analyses were performed to remove the effects of head motion (12 total motion covariates: 6 motion parameters plus temporal derivatives), physiological artifacts (10 total CompCor eigenvariates: five each from eroded WM and CSF masks), and artifactual scans. The resulting residual BOLD time series were band-pass filtered (0.01–0.1 Hz). Maps of normalized fractional amplitude of low-frequency fluctuations (fALFF) [45] were extracted at each timepoint. It has been previously shown that the amplitude of low-frequency fluctuations relates to spontaneous brain activity at rest [46]. We chose this methodological approach because it provides a straightforward solution to quantify and compare spontaneous brain activity in several separate resting state measurements.

### ROI definition

**Fig. 1:**
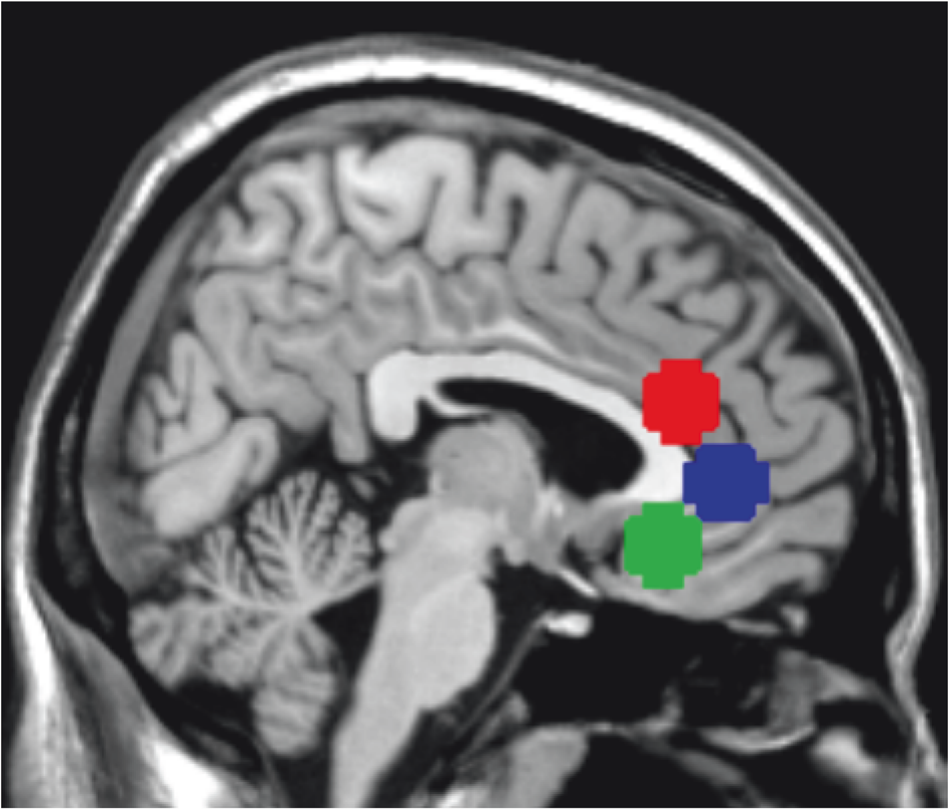
Selected regions of interest (ROIs) in the anterior cingulate cortex (ACC). Red = dorsal ACC. Blue = pregenual ACC. Green = subgenual ACC.

Based on the review article by Alexander et al. [4], the analysis focused on three regions of interest (ROIs) in the anterior cingulate cortex (ACC) that were built based on automated term-based meta-analyses performed on neurosynth.org. The ROIs were defined as spheres with a radius of 10 mm and the following center MNI coordinates: Subgenual ACC (sgACC; 0, 28, -12), pregenual ACC (pgACC; 0, 42, 2), and dorsal ACC (dACC; 0 32 20). The ROIs are shown in Fig. 1.

### Statistical analyses

Spontaneous brain activations in the predefined ROIs were analyzed using a mixed ANCOVA with the between-subjects factor *group* (PK, LK, PP), and the within-subjects factor *timepoint* (baseline, acute, delayed). Baseline activations were added as covariates into the model to control potential group differences before the infusion. Huynh-Feldt (HF) corrections were applied when necessary. Unadjusted paired comparisons and visualizations of the marginal means were conducted to interpret significant ANCOVA effects. Correlation analyses were conducted using Pearson’s correlation coefficient. All statistical analyses were conducted using SPSS version 27 (IBM, USA).

## Results

Seventy-five male and female subjects completed the fMRI procedures and were randomly assigned to one of the three treatment conditions in a 1:1:1 ratio. Due to insufficient data quality of the imaging data, the final sample for the analysis of the resting state data consisted of *N* = 70 subjects, of which *N* = 23 were in the placebo-placebo (PP) group, *N* = 23 in the placebo-ketamine (PK) group, and *N* = 24 in the lamotrigine-ketamine (LK) group (see Supplementary Fig. 1). Groups did not differ regarding age, sex, and trait negative emotionality (all *p* > 0.05). The subjective experience of dissociation and altered states of consciousness differed between groups. Both, the PK and LK groups had increased scores on the DSS scale and on the subscales of the 5D-ASC questionnaire. Detailed behavioral and demographic results are provided in Supplementary Table 1 and in our previous publication [28].

### fMRI results

**Fig. 2:**
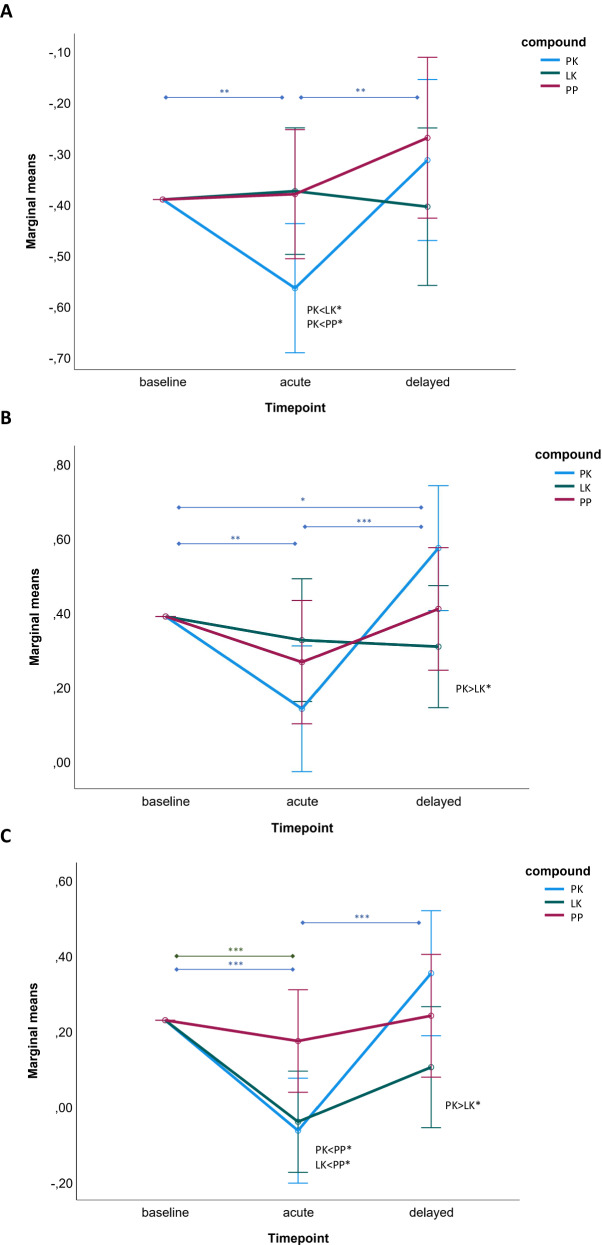
Estimated marginal means of the timepoint x compound interaction on spontaneous brain activity in subregions of the anterior cingulate cortex (ACC). **A** Subgenual ACC; **B** pregenual ACC; **C** dorsal ACC. The calculated ANCOVAs included the baseline activity of the respective subregion as a covariate. Stars depict the results of the calculated post-hoc comparisons. *p < 0.05; **p < 0.01; ***p < 0.001. PK placebo-ketamine group, LK lamotrigine-ketamine group, PP placebo-placebo group.

The mixed ANCOVAs calculated for the subregions of the ACC showed the following results: Spontaneous brain activity in the sgACC showed a significant effect of time (*F*(2, 132) = 14.21, *p* < 0.001, *η*_*p*_*²* = 0.18), a marginally significant interaction effect between time and group (*F*(4, 132) = 2.12, *p* = 0.084, *η*_*p*_*²* = 0.06), and no main effect of group. Paired comparisons showed that sgACC activity decreased from baseline to the acute timepoint in the PK group (*p* = 0.008) and increased from the acute timepoint to the delayed timepoint (*p* = 0.004). The LK and PP groups showed no significant effects between timepoints. At the acute timepoint the PK group showed lower sgACC activity compared to the LK (*p* = 0.036) and PP (*p* = 0.044) group. No other paired differences between groups were observed. The results for the sgACC are visualized in Fig. 2A.

For the pgACC, a significant effect of time (*F*(2, 132) = 10.16, *p* < 0.001, *η*_*p*_*²* = 0.13), a significant interaction effect between time and group (*F*(4, 132) = 2.75, *p* = 0.038, *η*_*p*_*²* = 0.08), and no main effect of group was observed. Paired comparisons showed that pgACC activity decreased from baseline to the acute timepoint in the PK group (*p* = 0.005), increased from the acute timepoint to the delayed timepoint (*p* < 0.001), and increased from baseline to the delayed timepoint (*p* = 0.032). The LK and PP groups showed no significant effects between timepoints. At the delayed timepoint, the PK group showed higher pgACC activity compared to the LK group (*p* = 0.03). No other paired differences between groups were observed. The results for the pgACC are visualized in Fig. 2B.

Brain activity in the dACC showed a significant effect of time (*F*(2, 132) = 7.82, *p* < 0.001, *η*_*p*_*²* = 0.11), a significant interaction effect between time and group (*F*(4, 132) = 3.05, *p* = 0.023, *η*_*p*_*²* = 0.09), and a marginally main effect of group (*F*(2, 66) = 2.53, *p* = 0.087, *η*_*p*_*²* = 0.07). Paired comparisons showed that dACC activity decreased from baseline to the acute timepoint in the PK (*p* < 0.001) and in the LK (*p* < 0.001) group. In the PK group, a significant increase from the acute to the delayed timepoint was additionally observed (*p* < 0.001). The PP group showed no significant effects between timepoints. At the acute timepoint lower dACC activity was observed in the PK group (*p* = 0.018) and LK group (*p* = 0.028) compared to the PP group. At the delayed timepoint, the PK group showed higher dACC activity compared to the LK group (*p* = 0.037). No other paired differences between groups were observed. The results for the dACC are visualized in Fig. 2C.

To exploratively investigate the main drug effect of ketamine on ACC activity, we conducted a voxel-wise analysis of the entire ACC (as defined by the neuromorphometrics atlas, Neuromorphometrics, Inc.) at the acute timepoint, and compared the activation patterns between the PK and PP groups. This analysis revealed three distinct clusters that closely resembled the ACC subregions that were defined as ROIs in the main analysis. The strongest activity reduction in the entire ACC was found in the region of the sgACC. Visualization of the clusters and statistics are shown in Supplementary Fig. 2.

### Correlation analysis

**Fig. 3:**
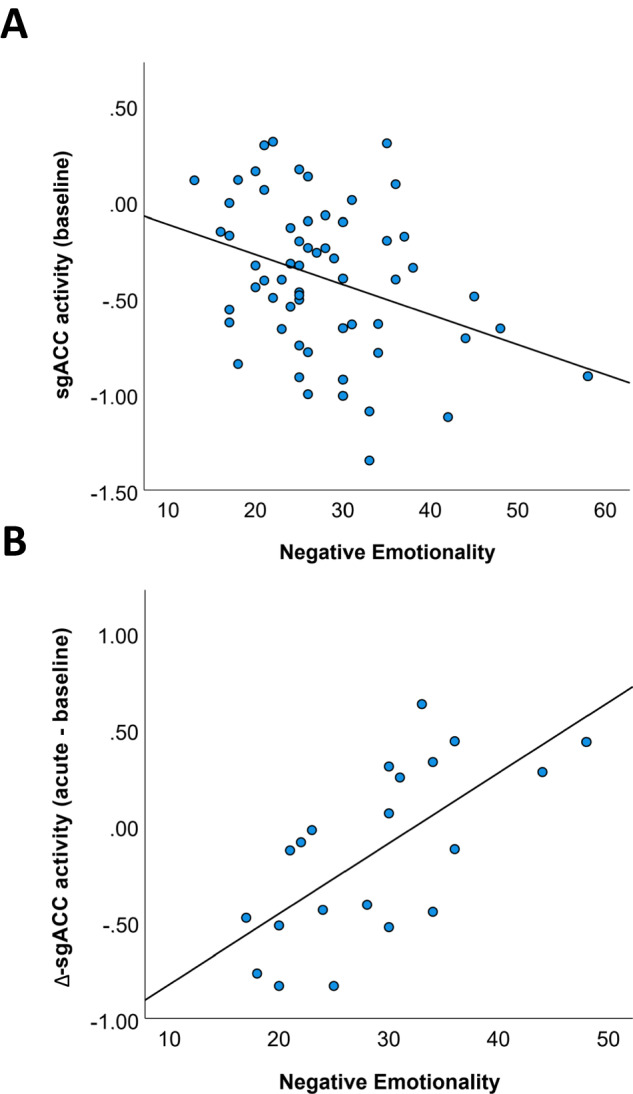
Association between trait negative emotionality and sgACC activity. **A** At baseline across all groups (*N* = 60); **B** sgACC activity changes (acute - baseline) in the PK group (*N* = 21).

To examine the relationship between ACC activity and negative emotionality, correlation analysis between baseline activity in the three subregions of the ACC and the negative emotionality score obtained from the BFI-2 were conducted for the whole sample. A significant negative relationship was observed for the sgACC (r(60) = −0.34, *p* = 0.008). Less sgACC activity at baseline was linked to higher negative emotionality (see Fig. 3A). Baseline activity in the pgACC and dACC was not linked to negative emotionality (all *p* > 0.1). Furthermore, the relationship between ACC activity changes at the acute timepoint (Δ-activity = acute - baseline) and negative emotionality was investigated in the three treatment groups. Again, the sgACC was the only ACC subregion that showed a significant positive relationship in the PK group (r(21) = 0.67, p < 0.001), and in the PP group (r(18) = 0.63, *p* = 0.005), but not in the LK group (r(21) = 0.03, *p* = 0.91). In the PP group, the relationship was clearly driven by two subjects with extreme values regarding their negative emotionality score. When these were removed, the significant relationship disappeared in the PP group (*p* = 0.45). In the PK group, an increase in sgACC activity was linked to higher negative emotionality, and a decrease in sgACC activity was linked to lower negative emotionality (see Fig. 3B).

Further correlation analyses were conducted to investigate the relationship between ACC activity changes and the subjective experience of dissociation and altered states of consciousness during the ketamine infusion. These analyses were conducted separately in the two groups that received ketamine (PK and LK). Neither of these groups showed significant correlations between acute activity changes in the three investigated ACC subregions and the subjective experience of dissociation, or in the five subscales of the 5D-ASC (all *p* > 0.1).

## Discussion

In this study, we investigated the effects of ketamine on spontaneous brain activity in subregions of the anterior cingulate cortex (ACC). Effects were compared between three treatment groups that were administered ketamine (PK), ketamine after lamotrigine pretreatment (LK), and placebo (PP). All subjects underwent fMRI scanning before, during, and 24 h after administration of the respective compound to investigate acute and delayed effects of ketamine on spontaneous brain activity. Trait negative emotionality was assessed to link observed ketamine effects in a healthy sample to findings in depressed patients.

In the subgenual region of the ACC (sgACC), our results showed a treatment-specific strong decrease in activity from baseline to the acute timepoint in the PK group, but not in the PP and LK groups. This result is consistent with previous findings showing decreased sgACC activity after ketamine, and that administration of lamotrigine before ketamine attenuates this effect [7, 8]. In comparison to previous studies, we used a different methodological approach to quantify spontaneous brain activation [45]. Thus, our study underlines the robustness of this effect. Extending previous research, our study shows that sgACC activity in healthy participants is restored to baseline levels 24 h after ketamine administration. No group differences were observed at the delayed timepoint. To our knowledge, this is the first study to investigate the acute and delayed effects of ketamine on spontaneous brain activity in healthy participants. Studies that investigated delayed effects were reporting functional connectivity changes [47], or were conducted in depressed patients [24, 27]. It should be noted that some of the above studies report changes in functional connectivity of the ACC after ketamine. In our study, we focused on spontaneous ACC activity, as this is a robust finding in the ketamine imaging literature that may be related to ketamine’s antidepressant mechanism (for a recent review, see [4]). The results of studies investigating functional connectivity during and after ketamine are less consistent. The main aim of our study was to gain a deeper understanding of the functional significance of the brain changes associated with ketamine. Therefore, we decided to use an established imaging marker and, in addition to replicating previous studies, to investigate the effects in the ACC subregions, the effects of lamotrigine, the effects of time, and possible associations with the trait of negative emotionality.

Our finding that lamotrigine attenuates the effect of ketamine on sgACC activity suggests that this effect is likely related to altered glutamate transmission. Altered glutamate transmission is a direct consequence of ketamine’s primary effect of NMDA receptor blockage and it is considered likely that a transient increase in glutamate neurotransmission is related to the antidepressant effect of ketamine [48]. Ketamine’s primary effect of NMDA receptor blockage, in turn, is closely related to the transient dissociative state after ketamine [49], and there is an ongoing debate about the role of dissociation in the antidepressant effects of ketamine [50]. Interestingly, our results did not provide evidence that the observed effect of ketamine on sgACC activity is linked to the subjective experience of dissociation; a relationship that has been reported in a previous study [7]. Furthermore, our results did not show an attenuating effect of lamotrigine on dissociation, which is in line with the findings of Abdallah et al. (2017). In summary, the results of our and other studies suggest that the effect of ketamine on sgACC activity is linked to altered glutamate transmission [7, 8], while a link between sgACC activity and dissociation remains controversial [7, 32, 33].

The rapid and short-term reduction in sgACC activity during ketamine administration is of great interest with regard to its antidepressant properties, because aberrant sgACC activity has frequently been associated with mood disorders [11], and with the processing and regulation of emotion [51]. However, most ketamine studies in depressed patients were not conducted during the administration. Hence the direct relationship between the acute effect in the sgACC and reduction of depressive symptoms is not well-established, even though Ballard et al. (2015) reported that decreased sgACC activity approximately 4 h after ketamine administration was linked to reductions in suicidal ideation. Conversely, one study in depressed patients measuring ACC activity during ketamine administration found an increase in sgACC activity that was linked to symptom reduction [13]. Whether this controversial finding is due to methodological differences, or to differential effects in healthy participants and depressed patients remains an open question.

To approach a possible discrepancy between the effects of ketamine in healthy individuals and depressed patients, we assessed the personality trait of negative emotionality in our study. Negative emotionality expresses a trait experiencing elevated negative emotions like fear, anger, or depression [42], and poses a risk factor for the development of depression [52]. Thus, it could be useful to bridge the gap between conflicting results in patients and healthy controls. Our results showed that participants with higher trait negative emotionality had lower sgACC activity at baseline. A link between sgACC activity and negative emotionality has been previously reported [53]. In this study, individuals with higher neuroticism scores showed stronger sgACC activity after emotional stimulation. This finding is in line with several findings observed in depressed patients [11]. Interestingly, we found a negative association between negative emotionality and sgACC activity in our study. This difference could be due to the fact that we examined the sgACC at rest and not after emotional stimulation. In the PK group, participants with low negative emotionality showed stronger sgACC activity reduction during ketamine compared to participants with higher negative emotionality scores. Participants with the highest negative emotionality scores even showed sgACC activity increases, a finding similar to the result found in depressed patients [13]. While this finding provides a potential explanation for the conflicting findings observed in healthy participants and depressed patients, it also raises the question of whether the effect of ketamine on sgACC activity is one-directional, or whether it should rather be seen as a regulatory process dependent on the baseline condition of the sgACC. It could be argued that ketamine-induced upregulation of sgACC activity in participants with high trait negative emotionality in our study and in depressed patients [13], is somewhat counterintuitive, as sgACC overactivity has been linked to depression [11]. However, the sgACC has also been suggested to play a crucial role in emotion regulation [51], and studies have linked higher baseline ACC activity in depressed patients to favorable treatment outcomes [54]. As mentioned above, it is also important to consider the condition under which sgACC activity is examined (rest vs. task). Thus, further research is needed to elucidate the exact role of sgACC activity in depression and the function of ketamine-induced sgACC activity alterations.

As for the sgACC, time-dependent modulations of the pregenual region of the ACC (pgACC) were observed only in the PK group. No significant reduction in pgACC activity at the acute timepoint was observed in the LK group. Thus, the observed effects in the PK group are likely driven by a similar glutamate-based mechanism as in the sgACC. Although the pgACC and sgACC are considered functionally distinct subregions of the ACC, both are involved in emotion processing [11]. Thus, based on our results, it is difficult to judge whether the effects observed in these two regions are independent. Of note, the observed associations with negative emotionality were exclusively observed in the sgACC. Interestingly, pgACC activity at the delayed timepoint was not only restored but slightly increased compared to baseline. Furthermore, the PK group had higher pgACC activity compared to the LK group at the delayed timepoint. This finding could give some insight into longer-lasting effects of ketamine, such as an increased metabolism in the respective brain region due to ketamine-induced synaptic plasticity [1, 55]. The results observed for the dorsal region of the ACC (dACC) showed that acute ketamine administration reduced activity in both the PK and LK group, which suggests that the effect in the dACC is driven by a mechanism that is less dependent on altered glutamate transmission. Stone and colleagues reported an association between NMDA receptor occupancy in the dACC and negative symptoms on the brief psychiatric rating scale [56]. Thus, the effect in the dACC might be closer to the dissociative properties of ketamine, although our results did not show such a relationship for dACC activity reductions. More research is needed to define the exact functional significance of reduced dACC activity during ketamine. An interesting approach for upcoming studies would be to consider the receptor architecture of the ACC subregions. For example, a cytoarchitectonic study by Palomer-Gallagher and colleagues [57] showed lower AMPA receptor density in the posterior region of the dACC investigated here.

The absence of associations between dissociative and neural effects during ketamine could be due to the relatively low dose of ketamine that was administered. However, subjective effects differed significantly from the placebo group in both groups that received ketamine, and the observed strong effects at the neural level suggest that even at low doses, ketamine induces significant brain changes. Nevertheless, upcoming studies should consider higher or even multiple doses of ketamine to achieve stronger dissociative effects, which could lead to additional insights into the relationship between the acute subjective and neural effects.

There are some limitations to this study. Although the sample size is relatively large for a pharmacological fMRI study with three different treatment groups, it should be noted that effects should be replicated in an independent large sample. Due to the given sample size, we decided to use rather liberal statistical thresholds to avoid the conduction of Type II errors. For instance, we decided to report marginally significant ANCOVA results (*p* < 0.1) along with the respective effect size. Noteworthy, medium effect sizes according to Cohen [58] were observed for marginally significant ANCOVA results. It should also be noted that unlike previous studies reporting acute ACC changes after ketamine administration [7], we did not apply an analysis approach that allowed an exact temporal localization of the effect. Instead, we compared a temporal average of a complete resting state scan shortly before ketamine administration to a temporal average of a resting state scan shortly after ketamine administration. On the one hand, this could be seen as a limitation. On the other however, the converging results between previous studies and our results demonstrate the robustness of the acute effects of ketamine on ACC activity. It is also important to note that while a link between depression and the trait of negative emotionality has been established, the state of being depressed goes beyond that trait. Therefore, conclusions drawn from healthy individuals with trait negative emotionality to depressed patients should be interpreted with caution. Nonetheless, considering trait negative emotionality in the investigation of ketamine’s neural mechanisms could provide important complementary insights, particularly if the recently proposed hypothesis that ketamine especially affects neural systems associated with trait negative emotionality holds true [36].

In conclusion, our study provides the first evidence that ketamine produces short-term activity reduction in the three investigated subregions of the ACC. The effects in the more ventral regions might have a distinct functional significance compared to the effect in the dorsal ACC, because inhibition of glutamate release by pretreatment with lamotrigine only attenuated the effects in the ventral regions. Investigation of trait negative emotionality showed to be a useful tool to link findings in healthy participants to potential antidepressant properties of ketamine, as a strong link between sgACC activity reduction and negative emotionality was observed. Taken together, our results extend the knowledge of the effects of ketamine on spontaneous brain activity in the ACC. To complement this knowledge, upcoming studies should conduct similar investigations in large patient samples. A detailed understanding of how ketamine alters brain activity in healthy individuals and patients, and how the effects are related to emotional personality traits, appears to be important to fully understand how ketamine acts on dysfunctional emotional processing, and exerts its rapid antidepressant effects.

### Supplementary information

Supplementary Table 1

Supplementary Figure 1

Supplementary Figure 2

Supplementary Figure Legends 1 & 2

## Data Availability

All data generated or analysed during this study are included in this article. Further enquiries can be directed to the corresponding author.
