# Supplementary material for: Negative emotionality shapes the modulatory effects of ketamine and lamotrigine in subregions of the anterior cingulate cortex
Source: Transl Psychiatry. 2024 Jun 18;14:258. doi: 10.1038/s41398-024-02977-x (PMC11189565; doi:10.1038/s41398-024-02977-x)
Supplement: Supplementary file 1 — Supplementary Table 1 [file 41398_2024_2977_MOESM1_ESM.docx]

**Supplementary Table 1**

*Baseline demographics and psychometric data for each group.*

|  | N | PK | LK | PP | Group statistic |
| --- | --- | --- | --- | --- | --- |
|  |  | *M (SD)* | *M (SD)* | *M (SD)* |  |
| Age | 70 | 28.65 (6.51) | 26.7 (5.06) | 30.45 (6.82) | *F*(2, 67) = 2.87, *p* = .064 |
| Sex (F:M) | 70 | 4:19 | 10:14 | 9:14 | *X²*(2, 70) = 3.75, *p* = .153 |
| NE | 60 | 28.76 (8.29) | 26.52 (8.1) | 27.72 (8.5) | *F*(2, 57) = 0.36, *p* = .701 |
| DSS | 70 | 2.73 (1.87) | 2.32 (1.4) | 0.69 (0.68) | *F*(2, 67) = 13.61, *p* < .001 *** |
| OBN | 70 | 16.93 (16.68) | 17.3 (15.7) | 1.58 (2.47) | *F*(2, 67) = 10.48, *p* < .001 *** |
| DED | 70 | 11.7 (13.31) | 11.81 (10.7) | 2.21 (3.93) | *F*(2, 67) = 8.09, *p* = .001 ** |
| VRS | 70 | 14.62 (13.29) | 10.3 (12.05) | 2.36 (4.05) | *F*(2, 67) = 6.64, *p* = .002 ** |
| AUA | 70 | 6.69 (12.44) | 4.58 (4.58) | 1.08 (2.04) | *F*(2, 67) = 3.12, *p* = .051 |
| VIR | 70 | 40.35 (22.84) | 41.33 (24.19) | 13.59 (14.27) | *F*(2, 67) = 13.08, *p* < .001 *** |

*Notes.* PK, placebo-ketamine; LK, lamotrigine-ketamine; PP, placebo-placebo; NE, negative emotionality; DSS, Dissociation-Tension-Scale; OBN, oceanic boundlessness; DED, dread of ego dissolution; VRS, visionary restructuralization; AUA, auditory alterations; VIR, vigilance reduction.
