# Supplementary figures and images for: Negative emotionality shapes the modulatory effects of ketamine and lamotrigine in subregions of the anterior cingulate cortex
Source: Transl Psychiatry. 2024 Jun 18;14:258. doi: 10.1038/s41398-024-02977-x (PMC11189565; doi:10.1038/s41398-024-02977-x)

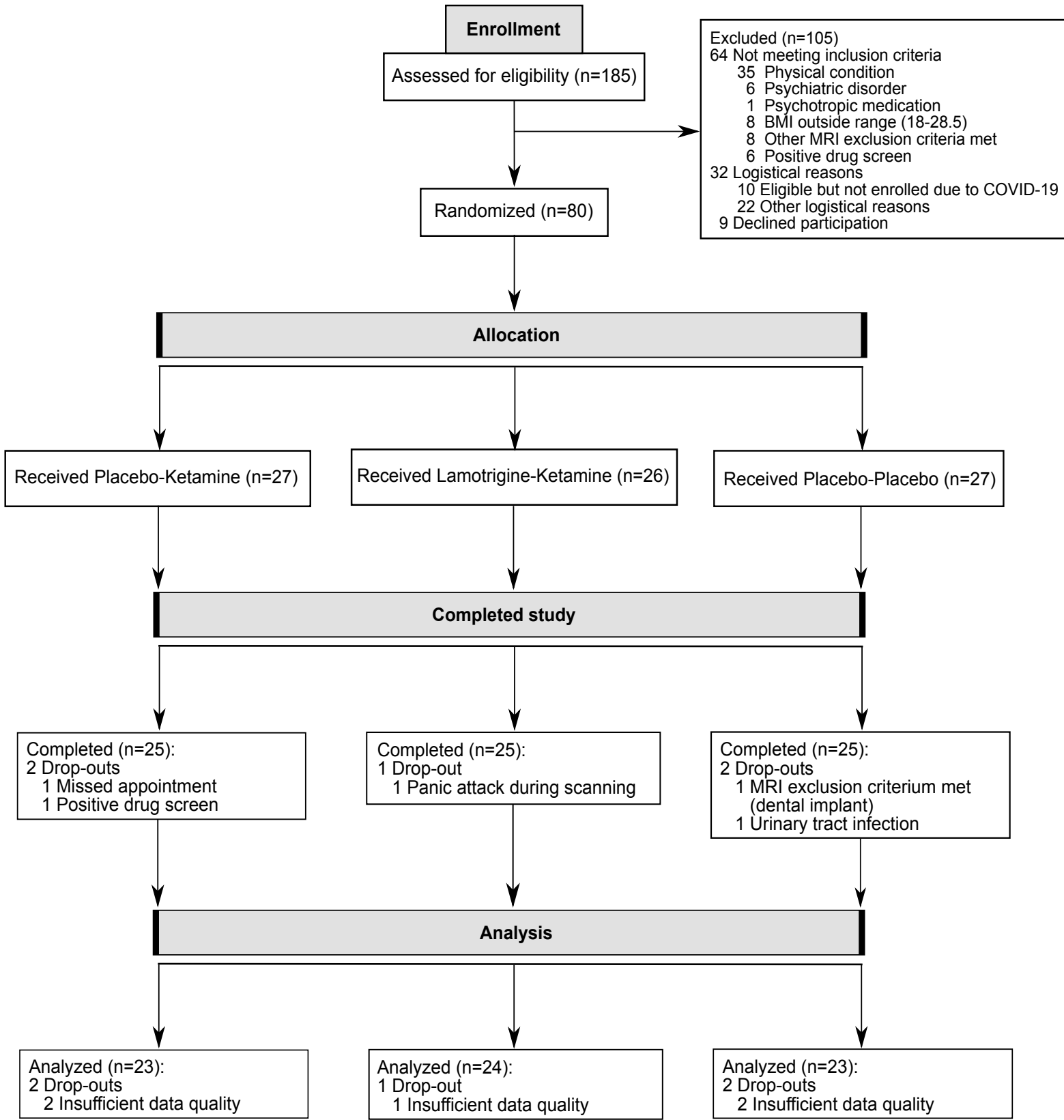

Supplement: Supplementary file 2 — Supplementary Figure 1 [file 41398_2024_2977_MOESM2_ESM.pdf]
