# Supplementary material for: Negative emotionality shapes the modulatory effects of ketamine and lamotrigine in subregions of the anterior cingulate cortex
Source: Transl Psychiatry. 2024 Jun 18;14:258. doi: 10.1038/s41398-024-02977-x (PMC11189565; doi:10.1038/s41398-024-02977-x)
Supplement: Supplementary file 3 — Supplementary Figure 2 [file 41398_2024_2977_MOESM3_ESM.pdf]

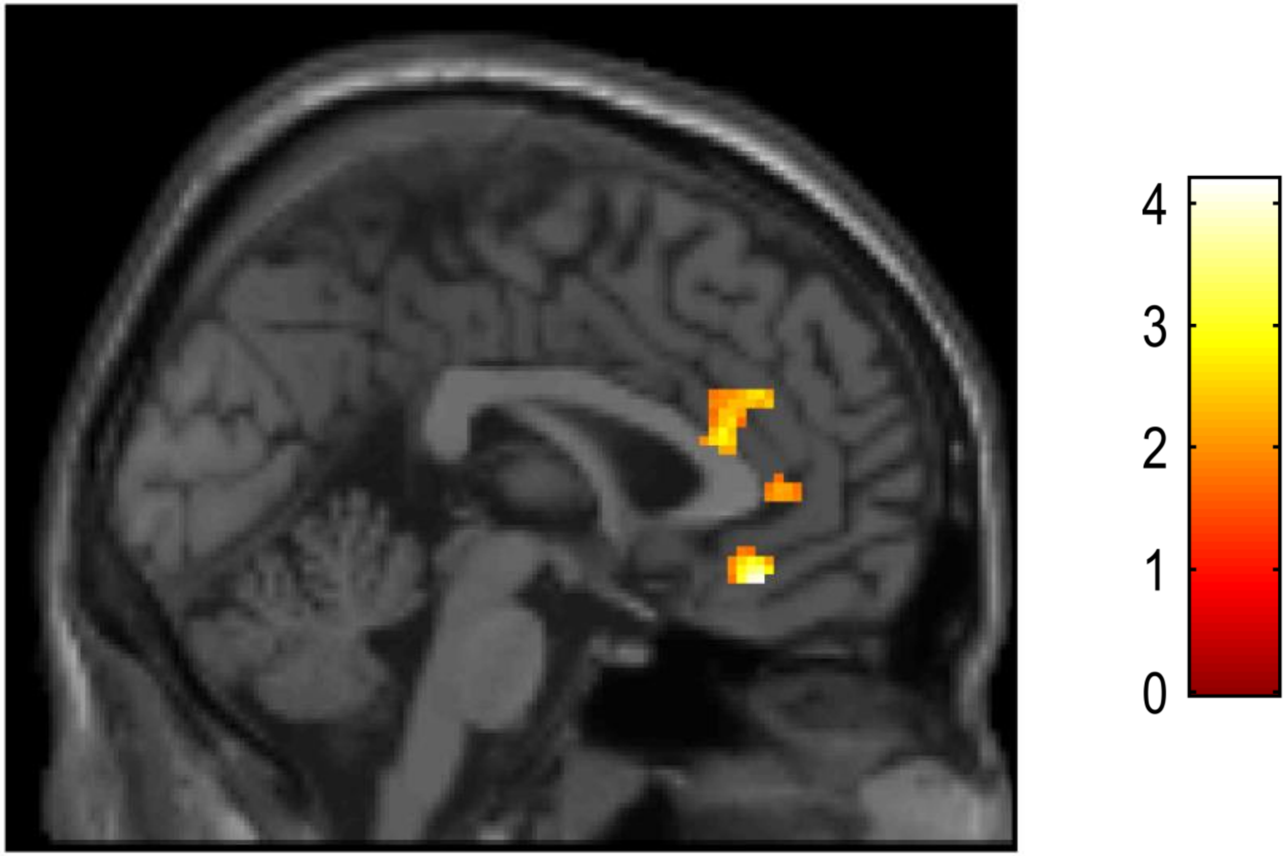


| Region | MNI center | Size | Peak-level statistics* | |
|---|---|---|---|---|
| | | | T | P |
| sgACC | -2 36 -14 | 74 | 4.18 | < 0.001 |
| pgACC | -4 26 18 | 77 | 2.77 | 0.003 |
| dACC | 4 40 6 | 319 | 3.44 | < 0.001 |

*Height threshold T = 1.68, Extent threshold k = 0 voxels
