# Supplementary material for: Negative emotionality shapes the modulatory effects of ketamine and lamotrigine in subregions of the anterior cingulate cortex
Source: Transl Psychiatry. 2024 Jun 18;14:258. doi: 10.1038/s41398-024-02977-x (PMC11189565; doi:10.1038/s41398-024-02977-x)
Supplement: Supplementary file 4 — Supplementary Figure Legends 1 & 2 [file 41398_2024_2977_MOESM4_ESM.docx]

**Supplementary Figure 1: Flow diagram.** This flow diagram shows the progress of participant exclusion and inclusion in the study**.**

**Supplementary Figure 2: Voxel-wise analysis of the entire ACC.** The figure shows differences in spontaneous brain activity between the PK and PP group at the acute time point. The identified clusters are the results of a voxel-wise analysis of the entire ACC (as defined by the neuromorphometrics atlas, Neuromorphometrics, Inc.). The table below the figure shows information about the cluster statistics and center MNI coordinates.
